# Variability of ultraplankton composition and distribution in an oligotrophic coastal ecosystem of the NW Mediterranean Sea derived from a two-year survey at the single cell level

**DOI:** 10.1371/journal.pone.0190121

**Published:** 2017-12-21

**Authors:** Maria Luiza Pedrotti, Laure Mousseau, Sophie Marro, Ornella Passafiume, Marjorie Gossaert, Jean-Philippe Labat

**Affiliations:** 1 Sorbonne Universités, UPMC Univ Paris 06, UMR 7093, LOV, Villefranche sur mer, France; 2 CNRS, UMR 7093, LOV, Villefranche-sur-Mer, France; Museum National d’Histoire Naturelle, FRANCE

## Abstract

Ultraplankton [heterotrophic prokaryotes and ultraphytoplankton (<10 μm)] were monitored weekly over two years (2009 & 2010) in a coastal area of the NW Mediterranean Sea. Six clusters were differentiated by flow cytometry on the basis of their optical properties, two heterotrophic prokaryote (HP) subgroups labelled LNA and HNA (low and high nucleic acid content respectively), *Prochlorococcus*, *Synechococcus*, autotrophic picoeukaryotes and nanoeukaryotes. HP represented an important component of the microbial assemblage over the survey with relatively small abundance variation through seasons. The carbon biomass ratio HP/ultraphytoplankton averaged 0.45, however this ratio exceeded 1 during spring. Ultraphytoplankton biomass made about 50% of the total autotrophic carbon estimates but this contribution increased up to 97% and 67% during the 2009 and 2010 spring periods respectively. Within ultraphytoplankton, nanoeukaryote represent the most important ultraphytoplankton group in terms of autotrophic carbon biomass (up to 70%). Picoeukaryote maximum abundance occurred in winter. *Synechococcus* was the most abundant population (maximum 1.2 x 10 ^5^ cells cm^-3^) particularly in spring where it represented up to 54% of ultraphytoplankton carbon biomass. The warmer winter-spring temperatures and the lengthening of the stratification period created a favorable situation for the earlier appearance of *Synechococcus* and its persistence throughout summer, paralleling *Prochlorococcus* development. *Prochlorococcus* was dominant over summer and autumn with concentrations up to 1.0 × 10 ^5^ cells cm^-3^. While the abundance of *Synechococcus* throughout survey was of the same order as that reported in western Mediterranean Sea, *Prochlorococcus* was more abundant and similar to the more typical oligotrophic and warm waters. The abundance variation of the ultraplankton components through the survey was relatable to variations in the hydrological and nutrient conditions.

## Introduction

In oceanic ecosystems, most organic carbon originates from atmospheric CO_2_ diffusing into surface seawater where it is directly metabolized by primary producers through photosynthesis. Phytoplankton play a major role as primary producers [1], contributing about 50% to the annual global net primary production [2] and form the base of marine food webs [3]. Different mechanisms that can be regrouped as carbon pumps, contribute to export part of this organic carbon to the mesopelagic and bathypelagic layers, down to sediment with variable sequestration times [4]. At any depth, organic matter is a source of CO_2_ through cell respiration, grazing and mineralization [5]. The efficiency and the intensity of the carbon pumps are ultimately determined by the balance between production, remineralization and food web processes [6]. Food webs differ across different trophic regimes. Under meso-eutrophic conditions large phytoplankton cells prevail over the small ones. In contrast, oligotrophic surface waters, essentially host small (≤ 2 μm) size phytoplankton favoured by better efficiency in nutrient uptake [7–8]. This dominance difference linking cell size and trophic regime is the basis of a trophic index [9] relating the relative abundance of eukaryotic pico and nanophytoplankton R_pn_, so that for instance R_pn_ >1 is indicative of an oligotrophic regime. Under oligotrophic conditions, less energy is transferred to higher trophic levels and a large fraction of primary production, up to 50%, is mineralised by heterotrophic prokaryotes (HP) [7, 10]. The planktonic community structure, especially the primary producers, and the microbial food web, play therefore a key function in the carbon cycle [11].

Among picoplankters, cyanobacteria are identified as the most ubiquitous marine phytoplankton group with *Synechococcus* being present in all marine systems and the genus *Prochlorococcus* being the most numerous photosynthetic organism on Earth colonising the euphotic zone of oligotropic oceans between 40°N and 40°S [12–14]. If the major role played by phytoplankton in the primary production and biogeochemical cycles is now well established [1, 15] the contribution of autotrophic picoplankton to the carbon cycle is larger than previously assumed. Picophytoplankton can also contribute to (i) supply newly synthesised organic carbon to micro-zooplankton, (ii) generate detritus and (iii) the sink of particulate organic carbon [16]. Moreover, besides being the dominant primary producers in oligotrophic oceanic waters, picophytoplankton may also play an important role in coastal waters [17].

The oceanic carbon cycle, particularly in coastal zones, is subjected to increasing anthropogenic pressure that is expected in the future to impact a greater number of taxonomic groups [18]. Thus, understanding the mechanisms by which organic matter is produced, transferred or exported, is essential to reach a predictive comprehension of the biogeochemical cycles and assess the effects of the climate changes and habitat degradation [19].

The NW Mediterranean Sea is considered as oligotrophic based on its low primary production mainly due to the very low concentration of inorganic phosphorus, particularly in summer [20–23]. At mesoscale, the hydrodynamics of the Ligurian Sea is characterized by alternating periods of mixing and stratification, yielding a strong seasonality in nutrient supply, primary production, phytoplankton and zooplankton community composition [24, 25]. Vertical water mixing is the main process that supplies nutrients to the upper layer. The Ligurian Sea coastal zones are characterized by a very narrow continental shelf bordering a deep (~2000 m depth) bottom, under the influence of open sea conditions and governed by the same pattern of nutrient limitation [26–28]. Survey on spatial and temporal patterns in community structure, revealed that the microbial food web is mainly composed by picoplankton and small flagellates (generally <5 μm) during most part of the year [29–30]. HP secondary production approaches 50% of primary production [31] and cyanobacteria and nanoflagellates are responsible for the major part of the primary production [32]. However, these studies were conducted during short periods and the dynamics of the picoplankton community were poorly or partially documented [33–36]. Moreover, their carbon dynamics at a seasonal scale were not addressed.

The present study is based on a 2-year time-series involving intensive sampling at the coastal Point B Station, in the Bay of Villefranche sur mer (NW Mediterranean Sea). The objective was to describe simultaneously the structure and composition variability of both ultraphytoplankton (<10 μm) and HP. We also addressed the nutrient availability and hydrological structures in order to determine how they can affect the vertical distribution of the microbial assemblages over seasons. This work will also contribute to document the abundance variability of the marine cyanobacterium *Prochlorococcus* that was not previously well detected in this coastal area.

## Materials and methods

### Study area and sampling

**Fig 1:**
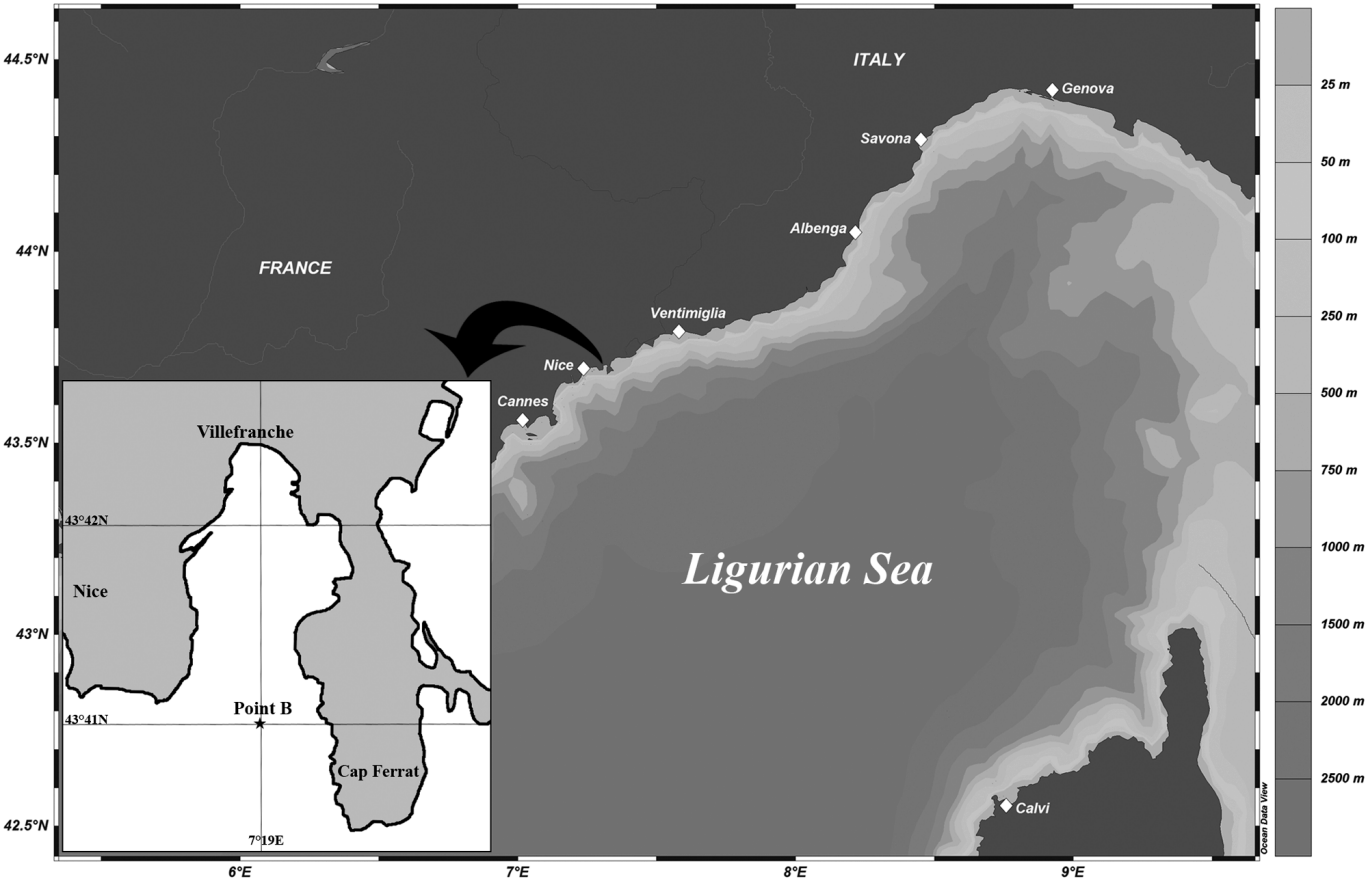
Location of the study area with the sampling station Point B (43°41.10 N, 7°19.00 E) in the Ligurian basin (Northwestern Mediterranean Sea). Schlitzer, R., Ocean Data View, http://odv.awi.de, 2016.

Data were collected during 101 weeks from January 2009 to December 2010 at Pt. B station (43° 41’ 10” N, 7° 19’ 00” E) NW Mediterranean Sea (Fig 1). The seawater was collected at 6 depths (surface, 10, 20, 30, 50 and 75 m) with Niskin bottles and placed in 10 dm^3^ polypropylene bottles. Sampling was conducted weekly between 9:00–10:00 am in order to limit short-term variability in group abundances. This site is routinely monitored since 1957 and the long-term series of physical and biogeochemical variables are gathered and maintained by the French national observatory network “Service d’Observation du Milieu Littoral” (SOMLIT) and the Observatory of Villefranche-sur-Mer. Since 1995 vertical distributions of temperature, salinity and fluorescence have been collected using a Seabird SBE25 autonomous profiler down to 80 m depth. Data where then processed using the Seabird program. Local meteorology was provided by Météo France station, the French national weather organization at Nice airport (43°38’54” N, 7°12’00” E, 2 m above sea level, about 5 km from Point B). It consists of daily measurements of precipitation (mm), wind speed (m. s^-1^) and solar radiation (J.cm^-2^). Hydrological data profiles of temperature, salinity and fluorescence of 2009 and 2010 were compared to a standard median year. The median values were calculated for each parameter from profile data of the last 17 years (1995–2008) in the same sampling station SOMLIT (http://somlit-db.epoc.u-bordeaux1.fr/bdd.php). In order to determine if 2009 or 2010 years are unusual or not, we calculated the anomaly of 2009 and 2010 to the median year.

### Nutrients, Chlorophyll a and particulate organic carbon

The inorganic nutrient concentrations nitrate and nitrite (NO_3_^-^; NO_2_^-^), and orthophosphate (PO_4_^3-^) were determined colorimetrically using a AA3 HR Seal Analytical auto-analyzer according to the procedures described by Aminot & Kerouel [37]. Total Chlorophyll *a* (T-Chl *a*) concentration was determined fluorometrically after filtering 1 dm^3^ seawater onto 25-mm Whatman GF/F glass fiber filters [38]. Particulate organic carbon (POC) concentrations were determined from samples taken at surface and 50 m depth. Samples (1 to 2L) were filtered onto pre-combusted Whatman GF/F glass-fiber filters and were analysed using a Euro EA Elemental Analyser. All these environmental measurements meet the quality control and protocols of SOMLIT (http://somlit-db.epoc.u-bordeaux1.fr/bdd.php).

### Flow cytometry

Sub-samples (2 ml) of seawater from each depth were immediately fixed in glutaraldhehyde (1% final concentration), flash-frozen in liquid nitrogen and stored at −80°C until analysis in the laboratory [39]. Single cell analysis was processed through a Becton Dickinson, FACSCalibur flow cytometer with a maximum flow rate of 65 mm^3^ min^-1^, equipped with a 488 nm argon laser. Each cell was characterized by 5 optical signals, forward and side scatter signals, green (530/30 nm), orange (585/42 nm) and red (> 670 nm) fluorescence. Data acquisition and treatment were processed with the CellQuest-Pro (BD-Biosciences) software. The sample flow rate was calibrated by adding to each sample fluorescent 1μm beads (Polyscience Inc., Europe) of known concentration. HP abundance was determined as described by Gasol and Del Giorgio [40]. Samples were diluted 2 -fold in autoclaved and 0.2 μm prefiltered TE buffer in order to avoid coincidence and then stained with (1:10000v/v) SYBR Green I (Molecular Probes). Two HP subgroups were resolved in green fluorescence versus side scatter cytogrammes and labelled LNA and HNA from their low and high nucleic acid content respectively according to Gasol et al. [41]. The abundance of autotrophic prokaryotes and pico- and nano phytoplankton was assessed from unstained samples following the method described by Marie et al. [42]. Four groups were resolved in red versus orange fluorescence cytogrammes, namely *Synechococcus*, *Prochlorococcus*, picoeukaryotes (< 2 μm) and nanoeukaryotes (2–10 μm) (S1 Fig).

### Biomass estimation

Chlorophyll *a* (Chl *a*) biomass and cell carbon content within each cluster were integrated over the 0–75 m layer by trapezoidal integration method [43]. The total autotrophic biomass was derived from a C: Chl *a* ratio-value of 81which was established by the linear relationship (y = 81.25 x + 88.47; p <0.001) between POC and Chl *a* concentration (203 samples from surface and 50 m depth). Our calculations based on samples from both the surface and deeplayers are approximately the average of the ratios of 125 [44] and 45 [45] at previously reported for the surface layer and the deep chloropyll maximum, respectively, for the same area.

HP carbon biomass was estimated using the conversion factor of 20 fg C cell^−1^ [46]. The conversion factors were 49 fg C cell^−1^ and 250 fg C cell^−1^ for calculating *Prochlorococcus* carbon biomass [47] and *Synechococcus* carbon biomass [48]. Pico and nanophytoplankton biomasses were estimated by using the equation of Verity et al. [49].

$$\text{C}(\text{pg})=\text{c}*{\text{biovolume}}^{0.866}$$ (1)

Biovolume in given in *μ*m^3^, c values are 0.405 and 0.239 for pico and nano eukaryotes respectively. These values were derived from the curve constructed by using the set of coeff-cell size [49]. The resulting carbon biomasses per cell are 1.393 and 14.133 pg C cell^−1^ for small and large eukaryotes, respectively.

### Statistical analysis

A Principal Components Analysis (PCA) was used to assess the relationships between microbial and ultraphytoplankton community structures and their hydrological environment. The dataset consisted of 101 week values, from January 6 2009 to December 28 2010, described by 42 biological active variables: concentrations (cells cm^-3^) of HP, *Synechococcus*, *Prochlorococcus*, autotrophic picoeukaryotes and nanoeukaryotes, Chl *a* (μg dm^-3^) and sample fluorescence (unit of fluorescence) at 6 discrete depths (0, 10, 20, 30, 50, 75 m). The nutrient ((NO_3_^-^; NO_2_^-^; PO_4_^3-^; Si(OH)_4_) at the same 6 depths and POC and PON concentrations (at surface and 50m) are illustrative variables transformed into rows (N = 7420). They were also projected in the factorial space, in order to improve interpretation of the results. The missing 8 values of the active data were estimated using two different methods: when a single value was missing between two existing ones, a linear interpolation was done. In the case where several successive values were missing an iterative approach using a PCA was performed. The multifactorial treatments were made using R language with FactoMineR and missDT libraries packages. The statistical significance of PCA axes was tested by the method of Boostraped data and the method of Broken-Stick model [50]. Moreover, potential relationships among integrated variables were tested by Spearman correlation coefficients (*p*). The intraset correlation coefficients of the different variables as well as the eigenvalues of axes were selected at significance level, P <0.05. Differences in POC, bacterial concentrations related to depth and to hydrological periods were tested by one-way analysis of variance (ANOVA) followed by a Post Hoc comparison using Tukey’s and least significant difference (LSD) test. Differences of ultraphytoplankton biomass between hydrologically defined periods were assessed by Kruskal-Wallis analysis of variance followed by a Mann-Whitney U-tests in case of significant differences.

## Results

### Environmental conditions

**Fig 2:**
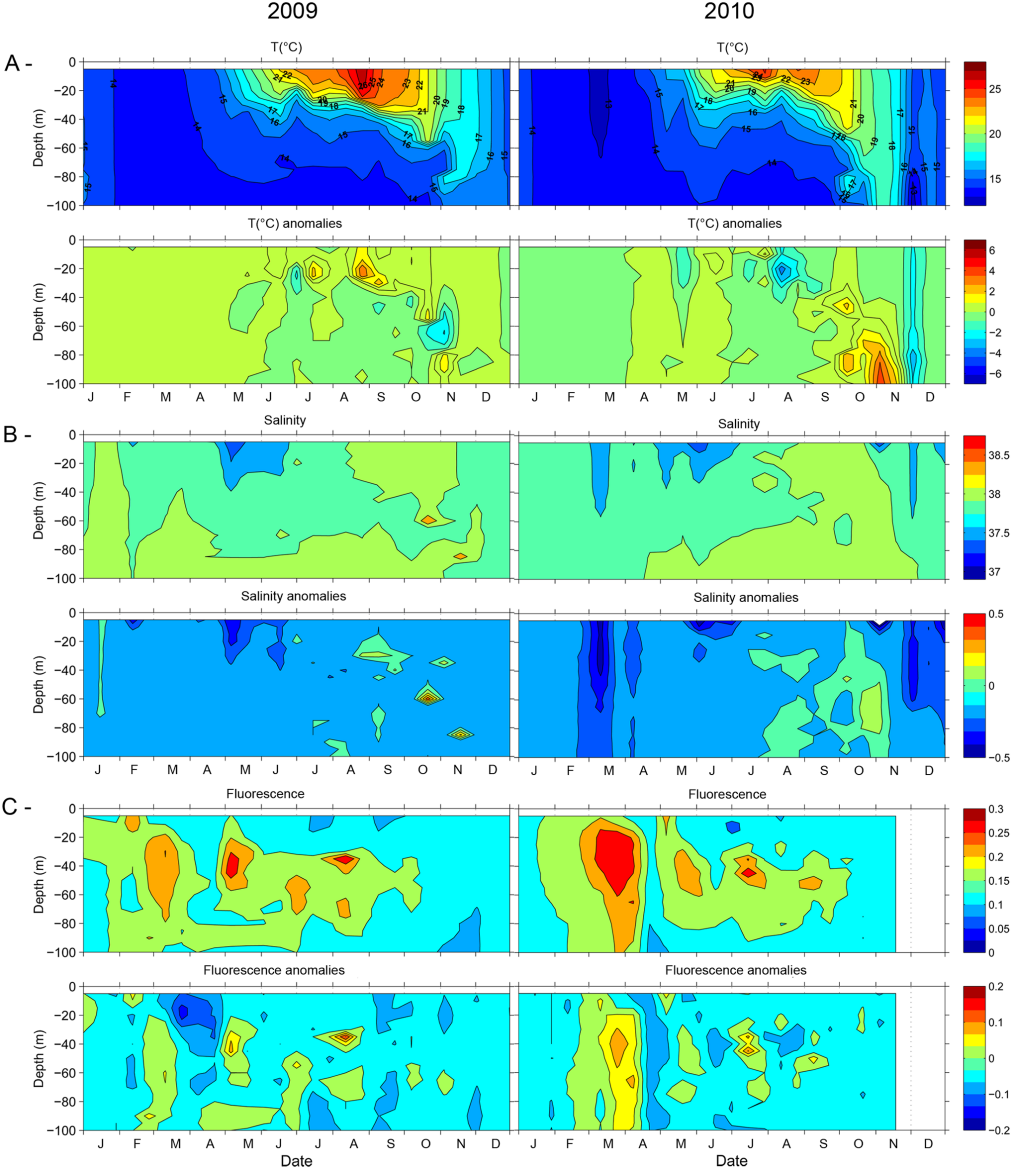
Vertical distribution of the physical variables collected weekly along the upper 75 m at Pt. B from January 2009 to December 2010. (A) Temperature (°C) and temperature anomalies, (B) Salinity (PSU) and salinity anomalies and (C) fluorescence (UF) and fluorescence anomalies. The anomaly graphs represent the standard deviation of a given year with respect to the median year values of 13 years (1995–2008) for each variable.

**Fig 3:**
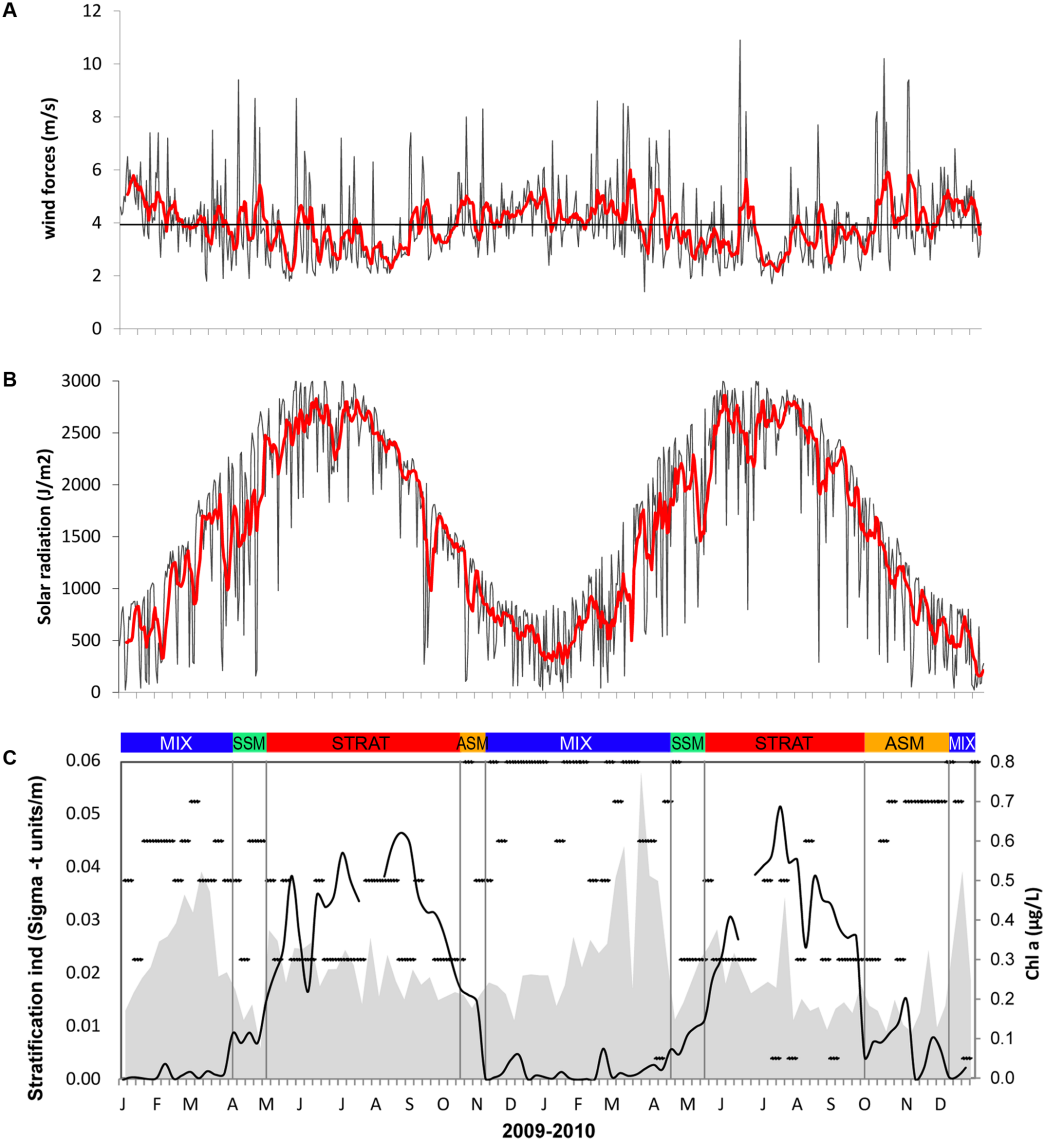
Meteorological and hydrological variables during the study period: (A) wind speed (ms^-1^); (B) solar radiation(Jm^2^) with were collected on a daily basis; red lines represent the weekly average. (C) The stratification index was calculated as the average density-excess difference between 0 to 75 m (Sigma-t kg m^-3^). MIX (mixed winter period), SSM (spring semi-mixed period), STRAT (summer stratified period), ASM (Autumn semi-mixed period). The transition from mixed to semi-mixed period was considered when there is a change of 0.05 in the stratification index and from semi-mixed to stratified period when the index reach 0.1. In gray, variations of integrated total Chl *a* (mg m^-2^) during the studied period. Dots represent the DCM depth (successively 0, 10, 20, 30, 50, 75 m).

The seawater temperature varied from 13 to 28°C between January 2009 and December 2010. In winter the average temperature through the water column was 14°C, with a minimum value of 12.5°C on March 9 2010 (Fig 2A). In 2009 temperature rose progressively until August, above 26°C at the surface, but only 23°C in summer 2010. The temperature then decreased through the autumn where the water column started to mix until winter of the following year. In 2009 water masses were warmer than in 2010 during winter and spring periods and exhibited higher profile-values during August and September. In 2009, temperature positive anomalies of +1 were observed from January to June and of +4°C in July and September indicating a warmer year compared to the average of the last 17 years (Fig 2A). In early November, the deep layer cooled, inducing negative anomalies as low as -2 to -4. In 2010, the summer period was shortened by a sharp decline of surface temperature in August, accompanied by an initial water column mixing, generating at 20 m depth a significant anomaly of -4 in August. Autumn was characterized by important changes in hydrological structure; the complete de-stratification of the water column occurred in early November. The rate at which this physical event occurred was also atypical, particularly in the deep water (100 m, temperature anomaly ca. +4). December was characterized by an important cooling with negative anomalies of -2 to -4 (Fig 2A). Salinity varied between 37.6 and 38.4 with lower values in surface waters in May 2009 and March 2010. Both years exhibited lower salinities compared with the last 14 years, except in September 2009 and during Autumn 2010. The lower salinities corresponded to negative anomalies varying from -0.5 to -0.2 for these periods (Fig 2B). Negative anomalies (-1 to -2) in fluorescence were observed in March and April 2009 between 0 and 50 m depth whereas a positive anomaly (+0.1) was observed in 2010 at the same period (Fig 2C). The maximum density (29) occurred in winter throughout the water column and in deep waters (80 m) during spring and summer of both years (data not shown). The average wind speed was 4 m.s^-1^ for both years. In 2009 the main wind event took place in mid-April with a maximum wind speed (9.4 m s ^-1^) reached on the 11^th^, whereas in 2010 the maximum wind speed (11.0 m. s^-1^) was observed on 10^th^ of June but other wind events occurred on early March (8.4 m. s^-1^) and in October and November (10.2 and 9.4 m.s^-1^, respectively) the latter being likely responsible for the de-stratification of the water column (Fig 3A). Atmospheric solar radiation increased gradually from the beginning of February reaching a maximal daily average value in June respectively 3086 and 3024 J.m^-2^ in 2009 and 2010. However, in 2010, the radiation increase was delayed compared to 2009, meaning that the spring reheating of the water column was also delayed, in line with the spring seawater temperature being lower than in 2009 (Fig 3B). The strongest rainfalls (148 and 447 mm in 2009 and 2010 respectively) were recorded in spring and autumn with a total of 34 and 30 raining days respectively.

In order to assess changes in the water column stability, a stratification index based on the density-excess difference between 75 m depth and surface was calculated according to Peterson and Bellantoni [51]. Results showed that in winter water masses are deeply homogenised and strongly stratified in summer, two-transition periods occurred in late spring (April-May) and late autumn. Given the pronounced differences in seasonality four main hydrological periods were set during the 2-year study: the winter mixed period (MIX) with colder and nutrient-rich water, the summer stratified period (STRAT) with thermally-stratified water column, and two semi-mixed periods in spring (SSM) and in autumn (ASM), the development of which differed with years (Fig 3C).

### Nutrients, Chl a and POC

Overall, nitrate concentrations were higher during winter (MIX) and spring semi-mixed (SSM) periods. For both years, the concentration decreased during summer, down to undetectable levels (< 0.05 μM). Orthophosphate concentration reached maximum values in January and April 2009 at 50 m depth respectively (0.38 and 0.45 μM). During the rest of the study period orthophosphate concentration was lower, varying between 0.20 and 0.008 μM. Silicate concentration was always > 1.0 μM with values up to 2.5 μM in 2009 at the end of February and November and in 2010 in March and July (S2 Fig). During the studied period, Chl *a* concentration ranged from 1.48 to 0.04 μg dm^−3^. In 2009, the highest values (0.74–0.6 μg dm^−3^) were observed in February-early March while in 2010, the highest Chl *a* concentration (0.8–0.7 μg dm^−3^) were found in March-April. This was reflected in the deep chlorophyll maximum (DCM), shallower in 2010 than in 2009 (Fig 3C). A second peak in Chl *a* concentration was observed at 30 m depth in late July 2009 and 2010 (1.48 and 0.87 μg dm^−3^ respectively), (S2 Fig).

Particulate organic carbon (POC) concentrations varied from 23.8 to 2 μM with an annual average of 10.4 and 8.3 μM at surface and 50 m respectively (data not shown). Lower concentrations were found in winter (December to February) and higher concentrations were determined at the end of March (23.2 μM) of both years and in June 2010 (19.1 μM). POC values were significantly higher at the surface than at 50 m depth during both years (ANOVA, F 5.9 and 6.11, p<0,01, N = 88 and 85).

### Seasonal-variations of ultraplankton-abundance

**Fig 4:**
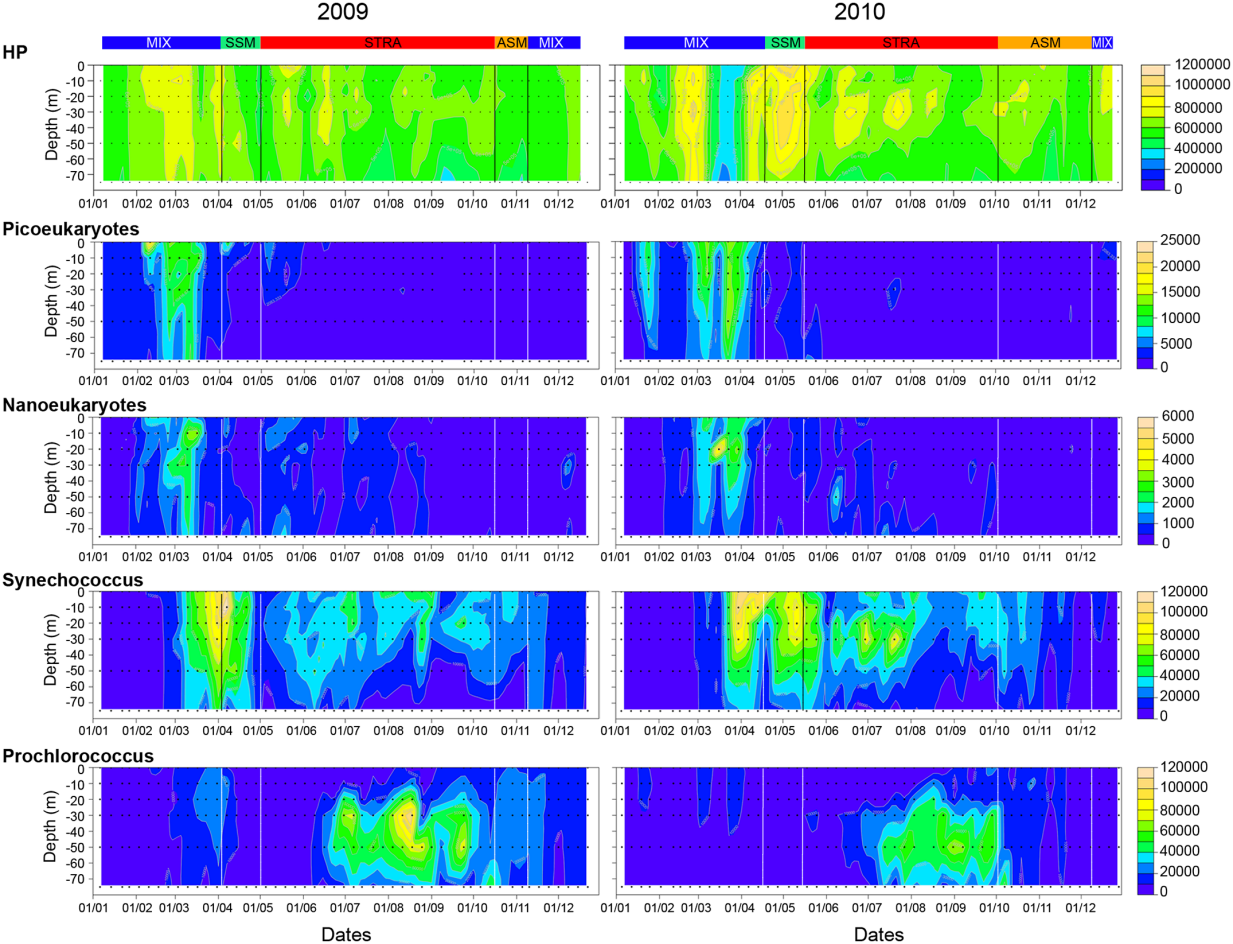
Vertical abundance (cells cm^−3^) of HP (Heterotrophic procaryotes) and ultraphytoplankton during the study period along the upper 75 m. Four autotrophic clusters are described namely *Prochlorococcus*, *Synechococcus*, picoeukaryotes and nanoeukaryotes. Black dots illustrate the 6-sampling depth. The coloured bars at the top represent the duration of the different hydrological periods (MIX SSM, STRAT, ASM) based on the calculated stratification index and on the ACP (see Methods for detail).

During the study period HP abundance ranged from 1.7 × 10^5^ to 1.1 × 10^6^ cells cm^−3^ with average values of 6.1× 10^5^ and 6.3 × 10^5^ cells cm^−3^ and related coefficients of variation (CV) of 15.1% (N 301) and 23.8% (N 308) in 2009 and 2010 respectively. All HP were resolved into HNA and LNA subgroups with the HNA > LNA in 2009 and 2010 (ANOVA, F 321.7 and 55.57, p <.0001) respectively. In 2009 HP and HNA abundances and integrated concentrations were positively correlated with T-Chl *a* concentration (p<0.001, N 301), while for 2010 no correlation was found (S1 Table). The overall abundances of HP significantly decreased with depth, (ANOVA, F 7.8, p<0.0001, N = 564). Post Hoc least significant difference (LSD) analysis revealed that HP concentration was significantly higher in the upper 30 m (< 0.0001) than at 50 m depth. At 75 m, their concentration was significantly lower than at the other depths (Fig 4). Heterotrophic Prokaryote abundances differed between hydrological periods (Table 1), Turkey HSD test (P<0.01) showed that HP concentration are higher during MIX and SSM periods respectively in 2009 and 2010.

**Table 1:** Heterotrophic prokaryote (HP), HNA cells concentrations (10^5^cells cm^−3^) and ratio of HP to total photosynthetic carbon biomass (HP/PHYTO) at Pt. B station in 2009 (N = 306) and 2010 (N = 300).

Variables	Year	Whole period			Hydrological periods				One-way ANOVA	
		Mean	Min	Max	MIX	SSM	STRAT	ASM	values	p
HP (10^5^ cells cm^−3^)	2009	6.2 (0.9)	3.3	9.2	**6.3 (0.8)**	**5.9 (1.0)**	6.0 (0.9)	5.5 (0.6)	F = 6.7	**
	2010	6.3 (1.5)	1.7	11	**6.3 (1.4)**	**8.6 (1.6)**	61 (1.0)	61 (0.8)	F = 14.2	***
HNA (10^5^ cells cm^−3^)	2009	3.6 (0.8)	1.9	5.7	4.2(0.7)	3.2(0.6)	3.3 (0.7)	3.3 (0.6)	F = 22.7	***
	2010	3.5 (0.9)	0.8	7.1	3.5 (1.2)	4.3 (1.0)	2.9(0.9)	2.6(0.6)	F = 17.8	***
HP/PHYTO ratio	2009	0.4	0.2	1.1	0.3	**0.5**	0.4	0.4	χ^2^ = 49.6	***
	2010	0.4	0.1	1.0	0.3	**0.6**	0.4	0.6	χ^2^ = 113.9	***

Mean ± SD, Min and Max values as well as the Mean values during hydrological events (MIX, SSM, STRAT, ASM) evidenced by the ACP (cfe Fig 3). F statistics apply to the ANOVA analyses, χ^2^ statistics apply to the Kruskal–Wallis test to identify the significant differences during hydrological events.

**P<0.001,

***P<0.0001.

In 2009, autotrophic picoeukaryotes appeared in the surface layer end of February (2.2 10^4^ cells cm^−3^) with average abundance remaining high up to mid-March (1.0 10^4^ cells cm^−3^). In 2010 they appeared in the surface layer end of January (25^th^) with abundance reaching 1.0 10^4^ cells cm^−3^. After a decrease in February their concentration increased again in the entire water column from March to the beginning of April, averaging 1.2 10 ^4^ cells cm^−3^. Marked declines were observed in both years for the following months with abundances < 1.0 10^3^ cells cm^−3^ (Fig 4).

In 2009, autotrophic nanoeukaryotes appeared mid-March with maximum abundance (3.4 10^3^ cells cm^−3^) at 10 m depth, then concentrations decreased in April to increase again in May (1x10^3^ cells cm^−3^). In 2010 higher abundances was observed from mid-March (5.4 10^3^ cells cm^−3^) down to 50 m until mid- April (2.0 10^3^ cells cm^−3^). For both years’ abundances then decreased to an average of 1.0 10 ^3^ cells cm^−3^ (Fig 4).

*Synechococcus* was numerically the dominant group of ultraphytoplankton during the 2-year survey, except in summer where its abundance was matched by that of *Prochlorococcus*. In 2009 average abundance during spring was 4.7 and 4.3 10^4^ cells cm^−3^ in March and April respectively. The vertical distribution peaked (1.2 10^5^ cells cm^−3^) at 10 m (7 April), and decreased with depth. In 2010 *Synechococcus* abundance reached high values in April and May (averages 4.5 and 5.4×10^4^ cells cm^−3^ respectively) and further increased in summer (8x10^4^ cells cm^−3^ at 30m). In autumn, the average *Synechococcus* abundance was 2.0 and 1.7 10^4^ cell cm^−3^ in 2009 and 2010 respectively with the lowest concentration (0.05 10^4^ cells cm^−3^) at 75 m depth on 5 November 2009. Over winters (December to February) *Synechococcus* abundance was low (average 0.5 10^4^ cells cm^−3^), (Fig 4).

Abundance of *Prochlorococcus* was higher during stratification period reaching maximum values between 30 and 50 m from July to September (Fig 4, Table 2). In 2009 maximum abundance of *Prochlorococcus* (1.0 10^5^ cells cm^−3^) occurred on 18 August at 30 m close to the nitracline and was shifted downward to 50 m (7.9 10^4^ × cells cm^−3^) in September to further decline (from 2.0 to 0.5 10^4^ cells cm^−3^) the rest of the year except end of March where a peak value of 3.0 10^4^ cells was observed. In 2010, *Prochlorococcus* was the major group from August to September with a maximum abundance (7.0 10^4^ cells cm^−3^) at 50 m depth on 31 August (Fig 4).

**Table 2:** Integrate biomasses (mg C m^-2^) over the 0–75 m water column of Chl *a*, HP, picoeukaryotes, nanoeukaryotes, *Synechococcus*, and *Prochlorococcus* at Pt. B station in 2009 and 2010.

Variables	Year	Whole period			Hydrological periods					
		Mean	Min	Max	MIX	SSM	STRAT	ASM	χ^2^values	p
Chlorophyll a	2009	2408	663	4698	**2714**	1416	2395	1797	21.7	***
	2010	2460	1050	6951	**3427**	1989	2124	1547	77.0	**
Picoeukaryotes	2009	211	20	1160	**355**	163	117	34	69.9	***
	2010	238	17	1354	**485**	223	99	64	163.2	***
Nanoeukaryotes	2009	594	45	2691	718	538	560	106	39.9	***
	2010	537	68	2174	**841**	406	465	177	82.5	***
*Synechococcus*	2009	357	67	1187	198	**681**	368	296	67.6	***
	2010	362	61	1087	271	**709**	435	251	45.2	***
*Prochlorococcus*	2009	69	4	230	40	21	**109**	79	68.2	***
	2010	48	6	138	25	15	**81**	42	70.7	**

Mean, Min, Max values are reported as well as the values during the main hydrological events (MIX, SSM, STRAT, ASM) evidenced by the ACP (cfe Fig 3). χ2 statistics apply to the Kruskal–Wallis test to identify the significant differences during hydrological events in 2009 (N = 306) and 2010 (N = 300). In case these Kruskal-Wallis tests were significant, Mann-Whitney U-tests were performed between data of pairs of hydrologically defined periods and are reported in bold.

Significance level, ** P<0.001,*** P<0.0001).

### Contribution to autotrophic biomass

The ratio of HP to total photosynthetic carbon biomass (HP/PHYTO) varied from 0.10 to 1.11 during the survey with an average value of 0.44 ± 0.17. HP/PHYTO remained <1, except on 28 April 2009 and 19 October 2010, where it was >1. Higher ratios were found in 2009 during the SSM period and in SSM and ASM period in 2010 (Mann-Whitney, P >0.001), (Table 1). During the study, the HP carbon biomass was in average 61 and 63% of that of autotrophic prokaryotes.

The carbon biomass integrated over the 0–75 m water column and assigned to Chl *a*, HP, autotrophic picoeukaryotes, nanoeukaryotes, *Synechococcus* and *Prochlorococcus* according to the main hydrological events and evidenced by the ACP is displayed in Table 2 and their contribution to total integrated autotrophic carbon in Table 3. The integrated autotrophic carbon-biomass derived from integrated Chl *a* concentration varied in the range 663–6951 mg C m^-2^ during the survey with maximum values found during winter mixed periods (Table 2). Picoeukaryote integrated carbon-biomass was significantly higher during MIX periods in 2009 and in 2010 respectivelly (Mann-Whitney, P >0.001 and P >0.001), (Table 2). They contributed up to 12% to the total autotrophic carbon biomass (Table 3). Nanoeukaryotes had similar carbon biomasses during MIX, SPRING and STRAT periods and lower during ASM period (Mann-Whitney, P >0.05) in 2009 and higher carbon biomass during MIX periods in 2010 (Mann-Whitney, P >0.05). They are the most important ultraphytoplankton group in terms of autotrophic carbon biomass reaching maximum values of 2691 and 2174 mg C m^-2^ in March of both years (Table 2). In ASM periods of both years nanoeukaryotes were minor contributors representing 6 to 11% of the autotrophic carbon biomass (Table 3). The contribution of *Synechococcus* to autotrophic biomass was significantly higher during the SSM periods of both years (Mann-Whitney, P >0.0001, Table 2) contributing to 44 and 35% of the integrated carbon respectively (Table 3). The integrated *Prochlorococcus* carbon biomass was significantly higher during the STRAT periods in 2009 and 2010 (Mann-Whitney, P >0.001, Table 2). In spite of being the most abundant group at this period; its contribution to the autotrophic carbon do not exceed 4,5%.

**Table 3:** Percentage contribution of picoeukaryotes, autotrophic nanoeukaryotes, *Synechococcus*, *Prochlorococcus* and Ultraphytoplankton to total integrated autotrophic biomass based on Chl *a* carbon and percentage contribution of picoplankton compartment (picoeukaryotes, *Synechococcus*, *Prochlorococcus*) to total Ultraphytoplankton biomass during the main hydrological events evidenced by the ACP.

Variables	Year	Whole period			Hydrological periods			
		Mediane	Min	Max	MIX	SSM	STRAT	ASM
Picoeukaryotes	2009	5.5	1	24.7	8.5	11.6	4.4	1.7
	2010	6.4	1	24.2	10	12	5	2
Nanoeukaryotes	2009	22.8	3.3	57.3	17.6	36.8	22.8	6.2
	2010	18.6	4.7	69.5	14.6	20.9	22.3	11.2
*Synechococcus*	2009	14.5	2.2	54.4	7.4	44.2	16.5	18.8
	2010	17.7	2.1	44.1	3.8	35.1	21.1	19.6
*Prochlorococcus*	2009	2.7	0.1	10.4	1.9	2.0	4.5	4.6
	2010	1.2	0.4	11.4	0.6	0.8	4.4	3.3
Ultraphyto/Phyto	2009	44.8	18.3	100	37.7	96.9	46.3	28.9
	2010	47.1	14.4	92.9	30.9	67.4	50.3	34.2
Pico /Ultraphyto	2009	24.9	12.9	68.7	19.5	58.8	25.3	25.6
	2010	25.9	6.7	53.1	15.5	47.7	29.1	24.9

Chl a was expressed in terms of carbon biomass by using the ratio C/Chl a = 81.

The ultraphytoplankton carbon biomass (Ultraphyto/Phyto) displayed high seasonal variability; during the SSM period it represents 97% and 67% of the total autotrophic carbon biomass in 2009 and 2010 respectively (Mann-Whitney, P< 0.01) (Table 3). During the SSM period the autotrophic picoplanktonic compartment (*Synechococcus*, *Prochlorococcus*, picoeukaryotes) represents 59% and 48% of the integrated carbon biomass of ultraphytoplankton (Pico/Ultraphyto) in 2009 and 2010 respectively (Table 3).

### Interannual variations and link with environmental parameters

**Fig 5:**
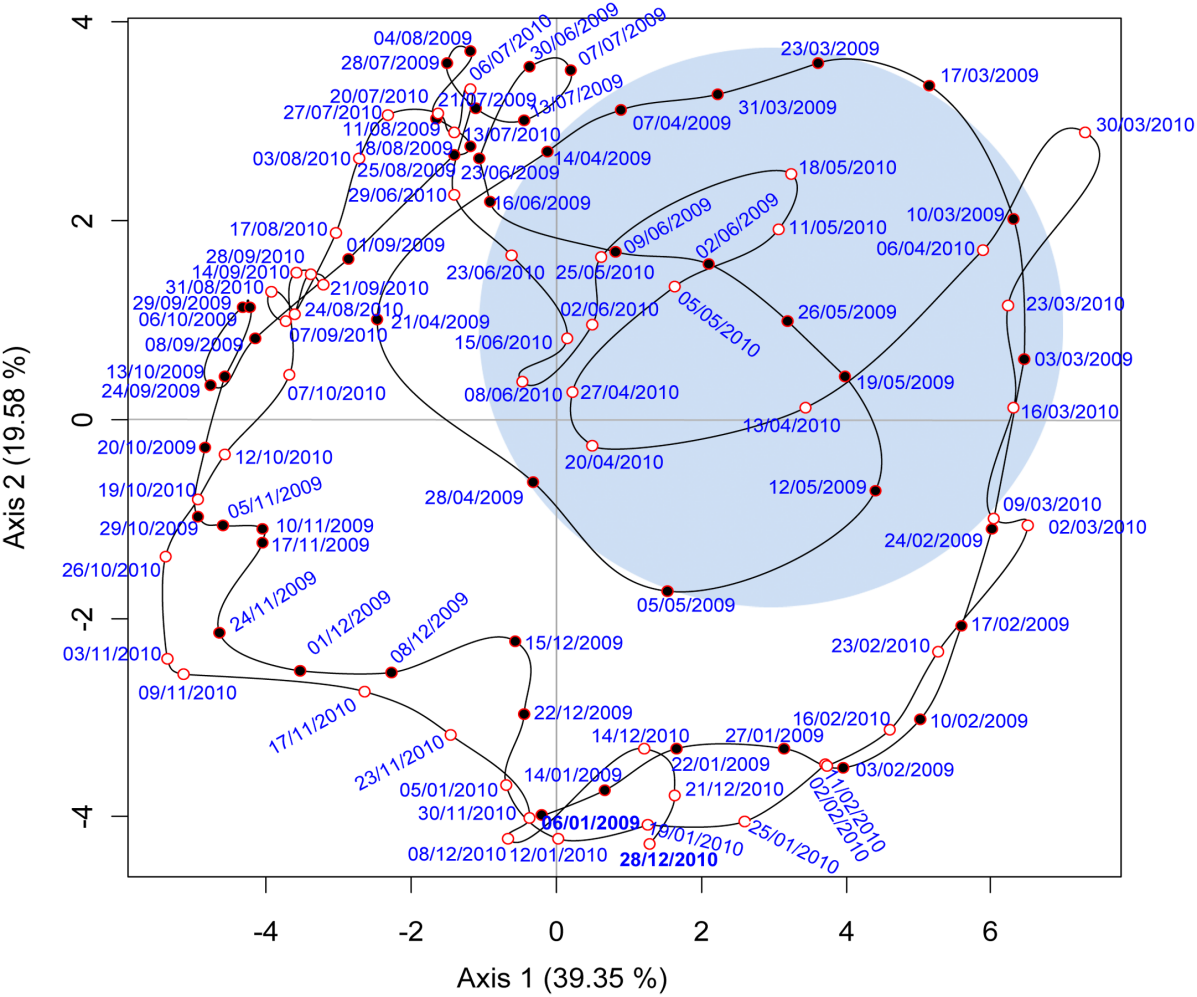
Principal Components Analysis (PCA) ACP-Plan 1/2, applied to data from 6 January 2009 to 28 December 2010. The 42-active data set consisted of 101 week values described by 42 biological variables: HP, *Synechococcus*, *Prochlorococcus*, picoeukaryotes, nanoeukaryotes abundances, fluorescence intensity and the Chl *a* concentration at 6 discrete depths (0, 10, 20, 30, 50, 75 m). Illustrative variables are concentrations of nutrients (NO_2_^-^, NO_3_^-^, PO_4_^3-^, Si(OH)_4_, POC concentrations. In red the year 2009, in blue 2010. N = 7420. The blue zone in the figure corresponds to the seasonal variability between 2009 and 2010 highlight by the ACP.

The results of the PCA variables representing the two sampling years are shown in the Fig 5. The first two axes accounted for 39.35 and 19.58% of the total variance with high statistical significance of the axes (N = 7420; p< 0.01) tested by the method of Boostraped data and Broken-Stick model [50], (S3 Fig). Axis 1 represents the spring semi mixed (SSM) communities contrasting with the autumn semi-mixed ones (September-October, ASM). Axis 2 represents stratified (STRAT) summer communities opposed to winter mixed ones (MIX). These results highlight (*i*) the significant variability between 2009 and 2010 in the hydrological features at the beginning of winter-spring transition period to late June (blue zone in the figure) and, (*ii*) the similarity in the environmental space from June to mid- March between the two years (Fig 5).

**Fig 6:**
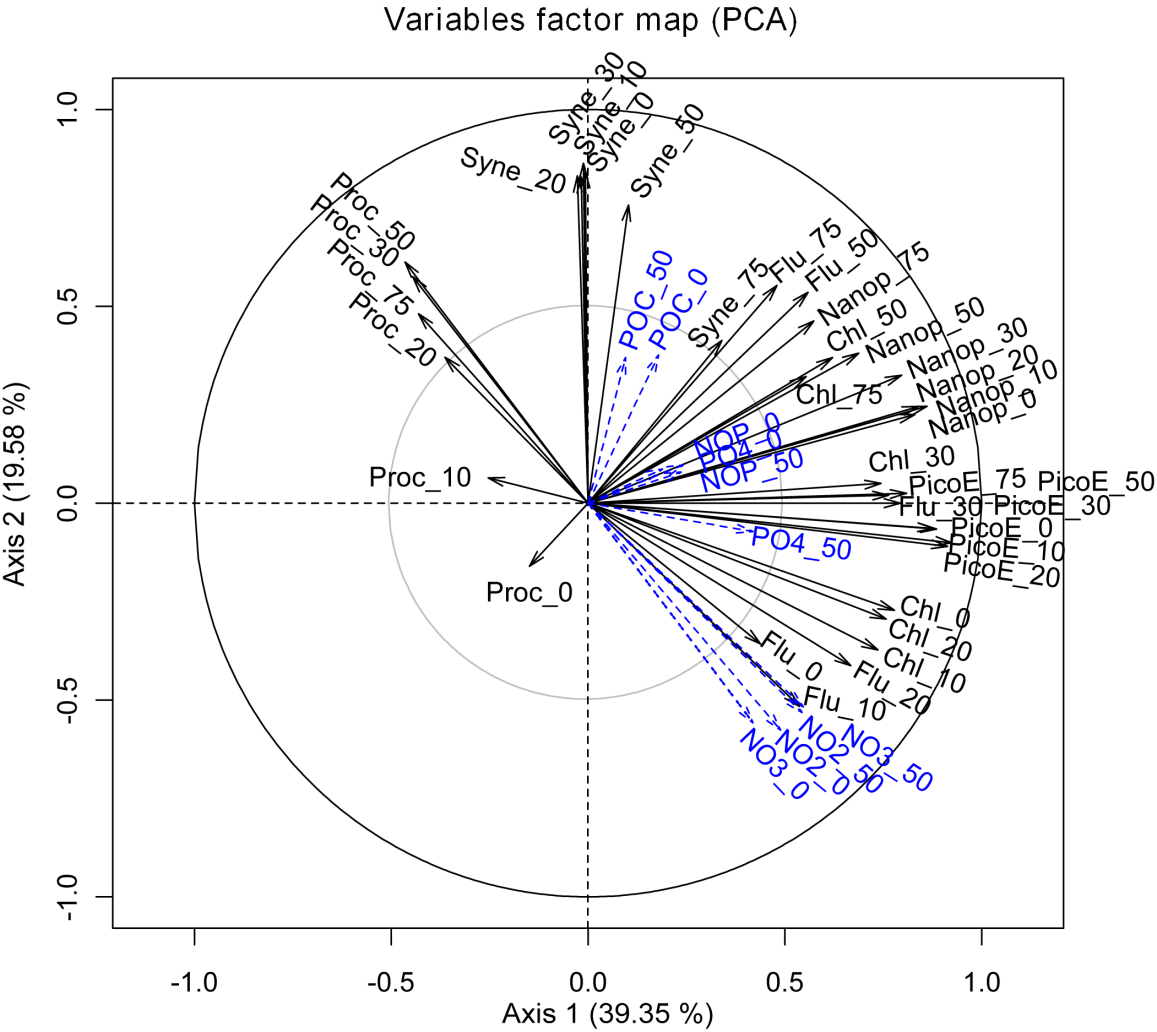
PCA variables factor map representing projection of variables on the plane defined by the first two principal components. The cycle of correlation represents the changes of the biological and biogeochemical parameters over time.

PCA variables projected in the first two factorial plan (Fig 6) decipher the temporal correlation and the vertical variability of biological and biogeochemical parameters. Moreover, in the S1 Table are given the results from Pearson correlation matrix among integrated environmental variables. For both years, the presence of nutrients from February to the end of March corresponds with an increase in the phytoplankton bloom indicator variables as showed by the positive correlations between nitrites and nitrates with fluorescence and Chl a (P < 0.001) (Fig 6, S1 Table). This relationship was associated with higher picoeukaryote abundances mainly in surface water as showed by the negative correlation with density in both years (*P <* 0.001), S1 Table.

In March 2009, a rapid transition occurred from spring bloom to a premature summer situation in April and a return towards a new spring situation state in May. The deepening of the maxima of Chl *a* concentration and fluorescence was accompanied by a downward shift towards 50 and 75 m of picoeukaryote and by nanoeukaryote maximum abundances (Figs 4 and 6). The stability of the water column and the increase in irradiance corresponded with an early stratification favoring the earlier development of Synechococcus that appeared in march at 75m and 50m and reached high concentration in surface water beginning of April. Nanokaryote developed again in May between 20 and 60 meters (Fig 4).

In 2010 a late cooling occurred in winter with an active mixing of the water column with an increase in chlorophyll concentration mid-March to mid-April (up to 0.8 dm^−3^, S2 Fig) corresponding to nanoeukaryote maximum concentration (Fig 4). The positive anomalies of fluorescence in 2010 and negative in 2009 match this transition period (Fig 2). In 2009 and 2010, during the STRAT period (from July to October) *Prochlorococcus* appeared in relative higher concentration of both years and was negatively correlated to chlorophyll, fluorescence, picoeukaryotes and nanoeukaryotes. In 2009, *Prochlorococcus* remain abundant until mid-November, in 2010 their concentration decreased one month before after a strong wind event (Figs 3 and 6). At the end of the autumn and during winter period (November to February), low values in Ultraphytoplankton abundance were observed.

The axis 3 and 4 (18.05% respectively of the variance) are two orthogonal structures representing HP abundance at all depths and the residual evolution in surface (0-20m) *Prochlorococcus* abundance (S4 Fig). Concerning HP, similarities were observed between both years: higher surface values in March and September-October and low values from the middle of April to June. The average abundance was higher in 2010 than in 2009. The additional data of *Prochlorococcus* surface abundance yielded a synthetic view of the *Prochlorococcus* dynamics at various depths: in the deeper layers (50–75 m), high values were observed in summer (July-August-September) (Figs 4 and 6), while at surface (0–10 m), two periods of abundance were observed, one in March and a second in October-November shifted in time compared to the depth peaks (S4 Fig).

## Discussion

### Variability in Ultraplankton distribution

The increasing survey effort conducted in pelagic waters during the last 25 years have improved our knowledge of the structure and dynamics of plankton communities in the open Mediterranean Sea [52]. Across the Mediterranean Sea, ultraphytoplankton is a large proportion of phytoplankton abundance and biomass [53, 54, 55] and according to seasons a major part of autotrophic carbon [56]. In the coastal area of NW Mediterranean Sea, current surveys have been mainly focused on microbial processes and metabolism [57,58] and long-term changes in zooplankton composition [59,60] often limiting phytoplankton analysis to that of Chl *a*. Our weekly survey over two years indicated that ultraphytoplankton was an important component of the autotrophic biomass in this coastal oligotrophic ecosystem, almost half the integrated total autotrophic biomass with proportions increasing up to 97% and 67% during spring of 2009 and 2010 respectively.

In both years, higher *Synechococcus* abundance was found during the SSM period and in summer, although this group was abundant throughout the year. This can be explained by the *Synechococcus* ability to respond quickly to such nutrient depletion. In the Mediterranean Sea, *Synechococcus* was shown to be a strong competitor for phosphate with an elevated P uptake rate [61,62]. The higher *Synechococcus* abundance in marine environments also stems from its ability to be photosynthetically competent at high light intensities [63]. Other nutrients can also be limiting factors for phytoplankton as silica that is assimilated by diatoms. When Si availability was low compared to N and P (episodic situations of Si limitation), a shift to flagellate-dominated communities was observed in the productive layer [64]. Experiments conducted in mesocosms showed that there was a 2.0 μM silicate threshold below which diatoms were outcompeted by flagellates, regardless the environment variability [65]. During our survey, silicate concentration was never < 1.0 μM, being probably sustained by the higher precipitation that characterized the whole study period. Indeed, important negative salinity anomalies were observed (Fig 2B). In the DCM layer, nitrates rather than silicates were the limiting factor with N/Si ratio between 0.3 and 0.9 during the mixed and semi mixed periods. The ability of these small ultraphytoplanktonic cells to better compete for nutrients under limiting conditions gives them an advantage over large cells [66]. This can partially explain the development of nanokaryotes in May 2009, likely replacing a microphytoplankton bloom.

The shift to more oligotrophic conditions was associated with an increase in *Prochlorococcus* abundance that dominated ultraphytoplankton in the 30-50m layers while *Synechococcus* persisted in surface waters. This maximum abundance above the nitracline can be explained by their more efficient uptake of recycled nutrients where *Prochlorococcus* may outcompete other algae [13, 67]. During the STAT period in 2009 *Prochlorococcus* remained abundant until mid-November, in 2010 their contribution decreased one month before after a strong wind event. The lengthening of the stratification period combined with environmental variables, notably temperature and light [22, 23] but also the stability of the water column appeared to be a requirement for their development and distribution range, as the population disappears with the breakdown of stratification. For the first time, we are able to decipher the annual distribution of *Prochlorococcus* in this near-surface oligotrophic waters. We have shown that *Prochlorococcus* exhibit a marked seasonal abundance cycle, varying approximately ten-fold between a winter minimum and a summer maximum. While the abundance of *Synechococcus* throughout survey was of the same order as that reported in western Mediterranean Sea [9, 67, 68], Prochlorococcus was more abundant than previously observed and their abundance vertical and pattern is similar to the one reported in more typical oligotrophic and warm waters [13, 53, 55, 67].

We showed that HP represents an important component of the microbial plankton in this area with the biomass exceeding autotrophic picoplankton biomass during the entire survey period. The carbon ratio of HP to autotrophic ultraplankton was similar to those observed in other regions of the Mediterranean Sea [69]. The amplitude of the HP-abundance seasonal-cycle through both years was relatively low supporting previous studies in the same location where bacterial production and respiration showed maxima following the primary production and Chl *a* maxima [57]. In temperate coastal ecosystems, the spring phytoplankton bloom is typically accompanied by a bacterial-production increase fueled by the DOC accumulation derived from primary production [70].

In systems where phytoplankton is the dominant source of labile organic matter, it is postulated that bacterial and primary productivity are tightly coupled [71]. Although the abundance of HP and HNA cells was positively correlated in our survey, only in 2009, there is a positive correlation with Chl a. Is likely that though strong correlations are not always observed because bacterioplankton populations are not only influenced by resource availability but also by losses predation and viral lysis and bacteria–bacteria interactions [72]. In 2010, un abrupt decline of HP abundance that occurred in March is probably responsible for this lack of correlation. This event was accompanied by a decrease in salinity, shown by the strong negative anomaly of surface to 50 m, and an increase in the concentration of nitrite and silicate. This follows probably from fresh water supplies since this area is under the influence of runoff of rainwater and outflows of local coastal rivers (Var, Arno and Roya), mainly in winter and spring when snow melts in the Alps [73]. This decline affected mainly the HNA cells fraction perhaps more likely to respond to environmental changes. Many studies have associated this subgroup with the most active members of the bacterial community responsible for the most important part of the bulk activity and generally linked to variables following the productivity index of the ecosystem [17, 74]. Conversely in Mediterranean Sea the activity in this subgroup was found to be poor predictor of ecosystem properties [75, 76]. It was also show that in oligotrophic systems LNA may have low cell specific metabolic activity [77]. The larger variability of HNA abundance (CV 77%) compared with that of LNA (CV 57%) may suggest the existence of a relatively constant pool of LNA cells in our survey. This question is still a matter of debate, phylogenetic analysis of HNA and LNA clusters [78] suggests that they were composed of the same dominant species with a wide range of physiological states resulting in cells with very different apparent nucleic acid contents. Interestingly, the relationship found between abundances of HP and *Prochlorococcus* in surface water shown by the ACP (S3 Fig) might reflect a common controlling factor such as grazing pressure or nutrient limitation as bacteria also require substrates other than carbon, e.g. those containing nitrogen and phosphorus concentration [7].

### Interannual variations and link with environmental parameters

Coastal areas are complex ecosystems, hydrology and winds can lead to changes in nutrient availability altering species competition, the microbial assemblage and the associated carbon cycle. The ACP highlighted a significant interannual variability in the microbial community structure during MIX period that likely resulted from the hydrological features. Although end of February of both years corresponded to the onset of the phytoplankton bloom, the year 2009 was characterized by warmer winter-spring temperature compared to 2010 and to the median year defined by the 1995–2008 dataset (Fig 2). These conditions likely affected the intensity of the mixing period, the transition towards spring conditions and the length of the stratification period. We can hypothesize that when the light and the stabilization of the water column became optimal for the development of larger cells, the increase in temperature did not enable a sustainable turbulent mixing and nutrients became depleted or at low levels. This has benefited ultraphytoplancton populations that have dominated the autotrophic biomass with a ratio ultraphytoplancton/Phyto by 97% over the SSM period (Table 3). The strong negative fluorescence anomaly observed in surface water between March and April 2009. (Fig 2) support this explanation. Moreover, a 2-month high frequency phytoplankton survey in the period using an automated flow cytometer did not detect either significant development of larger cells after the major increases in picoeukaryote abundance [36]. In 2010 the increase in nanokaryotes in March-April corresponded to the nanoplanktonic bloom previous observed in this area under similar condition. Based on pigment analysis [79], it was shown that under low nutrient conditions and low irradiation, the spring bloom was composed at 80% by chromophyte nanoflagellate with diatoms present in deeper layers mainly in August–September. In autumn, upon the stratification break down, superficial water is supplied with nutrients generating a second favorable period for bloom development as previous observed in this area [79, 80]. However, these conditions were not achieved in our survey; in 2009 the stratified period lasted longer with temperature remaining high (17°C) until December. Therefore, nitrate concentration remained low (< 0.5 μM), orthophosphate was undetected and no increase in chl *a* concentration (0.3μg dm^-3^) was observed. In 2010, destratification took place early October triggered by a strong wind event (maximum wind speed of 10.2 m s^-1^) but the water column re-stratified by mid-November and no increase in chl *a* was observed at this period.

### Consequences for the trophic food web

It was hypothesized that environmental changes affecting the structure and composition of phytoplankton biomass may disrupt links between primary producers and higher trophic levels, impacting the carbon cycle in coastal environment [81]. Anthropogenic stressors can impact the inputs of freshwater and atmospherical and terrestrial inorganic nutrient in the euphotic zone, modifying phytoplankton dynamics [82]. The Mediterranean Sea is considered as one of the most affected areas by global warming and the ecological response to temperature increase and changes in nutrient concentration could be a shift from a diatom-dominated ecosystem towards non-siliceous species and a regenerative system [22]. In the open sea DYFAMED station (NW Mediterranean), a 10-year time-series based on pigment analysis provided the first evidence of temporal changes in phytoplankton community [24]. Results revealed the dominance of small-size cells (picoplankton and nanoflagellates) with a decline of diatoms in periods of poor mixing [24]. Another time-series in the Bay of Calvi suggested that weak mixing combined with temperature increase and salinity decrease favours picoplankton and flagellate development [83]. It has already observed in this area that microphytoplankton blooms are of short duration or weak amplitude [79, 80]. If the frequency of hydroclimatic changes increase, especially spring temperature anomalies, this could have an important impact on phytoplankton bloom development. In a niche space models using projected future climate conditions, ocean warming may lead to shifts in both the distribution and composition microbial communities, for example, the distribution and abundance of *Prochlorococcus* and Synechococcus is predicted to increase especially in warmer oligotrophic areas [84]. In our survey, the high proportion of ultraphytoplankton within the autotrophic compartment, the extended *Synechococcus* seasonal cycle and the high *Prochlorococcus* abundance over summer and autumns not documented before, are indicators that need to be monitored to assess putative changes in coastal areas. In a study analyzing plankton successions Romagnan et al. [85] showed that top-down grazing by gelatinous filter feeders and predators might control the development of coastal phytoplankton bloom. The trophic interactions likely influence the entire plankton dynamics. The increase in gelatinous zooplankton abundance observed in NW Mediterranean Sea over a 30-year survey was attributed to a trophic reorganization induced by an oligotrophization due to warmer temperatures and lower water column mixing [25]. This shift favouring filter-feeding zooplankton over selective feeders such as copepods may contribute to a progressive change in the structure of the entire pelagic food web in the Mediterranean Sea [86]. Additional studies need to be accompanied by complete and simultaneous survey on microbial and higher trophic webs to further examine the variability in phytoplankton community and to assess the consequences of this changing features on carbon cycle in coastal environment. High frequency monitoring probably holds the key for understanding the oceanic carbon cycle and its feedbacks on climate change.

## Supporting information

S1 Fig Resolution by flow cytometry of heterotrophic prokaryotes (HP) and ultraphytoplankton assemblage composition during the study period. (A) Two subgroups of LNA and HNA bacteria were resolved in green fluorescence (FL1) versus side scatter cytogrammes (SSC) and labelled from their low and high nucleic acid content respectively (B,C) display cytograms of red fluorescence (chlorophyll a) versus side scatter resolving four cell groups: Synechococcus, Prochlorococcus, picoeukaryotes and nanoeukaryotes. Panel (C) display settings specifically to resolve Prochlorococcus population. In order to separate the population from the background noise we used a FL3 PMT at 650V and a gain of 1 while for the other autotrophic cells we used a FL3 PMT at 450V and gain of 1. The beads are the 1μm Trucount calibration beads (Beckton Dickinson).
(TIF)

S2 Fig Chemical characteristics of the water column from January 2009 to December 2010 for the upper 75 m. Nitrate (μM NO_3_^-^), orthophosphate (μM PO_4_^3-^), Silicates (μM Si(OH)_4_) and total Chl *a* (μg dm^-3^).
(TIF)

S3 Fig Principal Components Analysis (PCA) plots of Eigenvalues of correlation matrix. (TIF)

S4 Fig PCA variables factor map represents projection of variables on the plane defined by the axis 3 and 4 (18.05% respectively of the variance). The two orthogonal structures representing HP abundance at all depths and the residual evolution of surface (0-20m) *Prochlorococcus* abundance.
(TIF)

S1 Table Spearman correlation matrix among integrate variables for 2009 and 2010 N = 50. The threshold for significant correlation coefficients were *** P< 0.0001 and relevant (p≥ 0.50); ** P < 0.001 at p = 0.42; * P < 0.05 was at p = 0.23.
(DOCX)
