# Supplementary figures and images for: Variability of ultraplankton composition and distribution in an oligotrophic coastal ecosystem of the NW Mediterranean Sea derived from a two-year survey at the single cell level
Source: PLoS One. 2017 Dec 21;12(12):e0190121. doi: 10.1371/journal.pone.0190121 (PMC5739496; doi:10.1371/journal.pone.0190121)

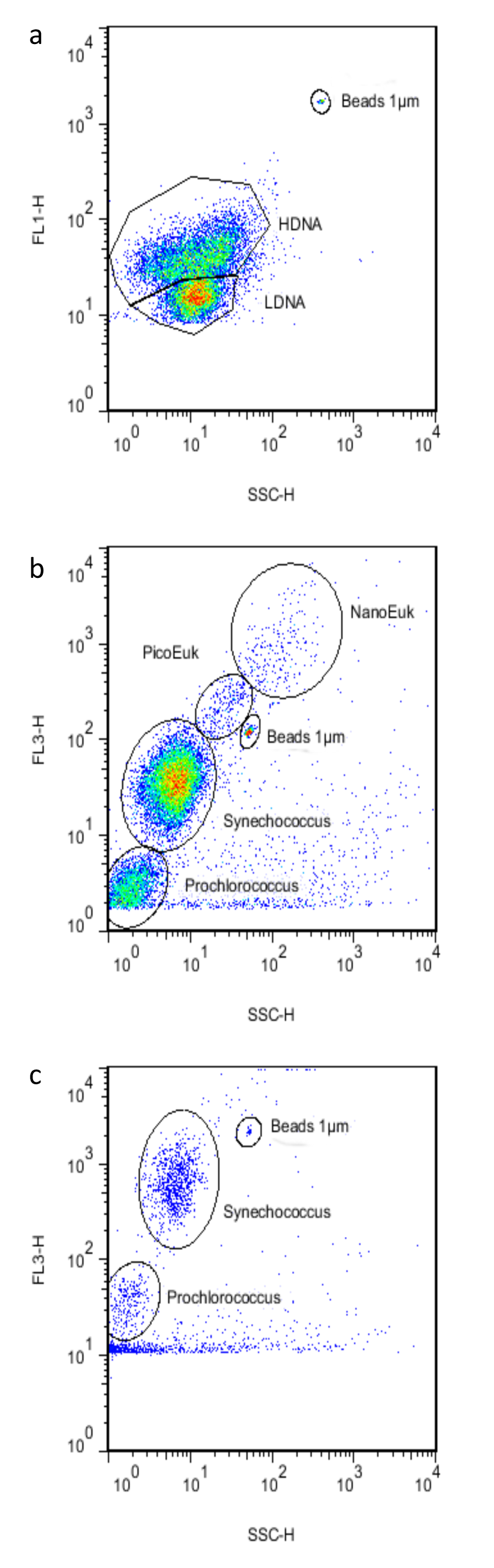

Supplement: S1 Fig — (A) Two subgroups of LNA and HNA bacteria were resolved in green fluorescence (FL1) versus side scatter cytogrammes (SSC) and labelled from their low and high nucleic acid content respectively (B,C) display cytograms of red fluorescence (chlorophyll a) versus side scatter resolving four cell groups: Synechococcus, Prochlorococcus, picoeukaryotes and nanoeukaryotes. Panel (C) display settings specifically to resolve Prochlorococcus population. In order to separate the population from the background noise we used a FL3 PMT at 650V and a gain of 1 while for the other autotrophic cells we used a FL3 PMT at 450V and gain of 1. The beads are the 1μm Trucount calibration beads (Beckton Dickinson). (TIF) [file pone.0190121.s001.tif]

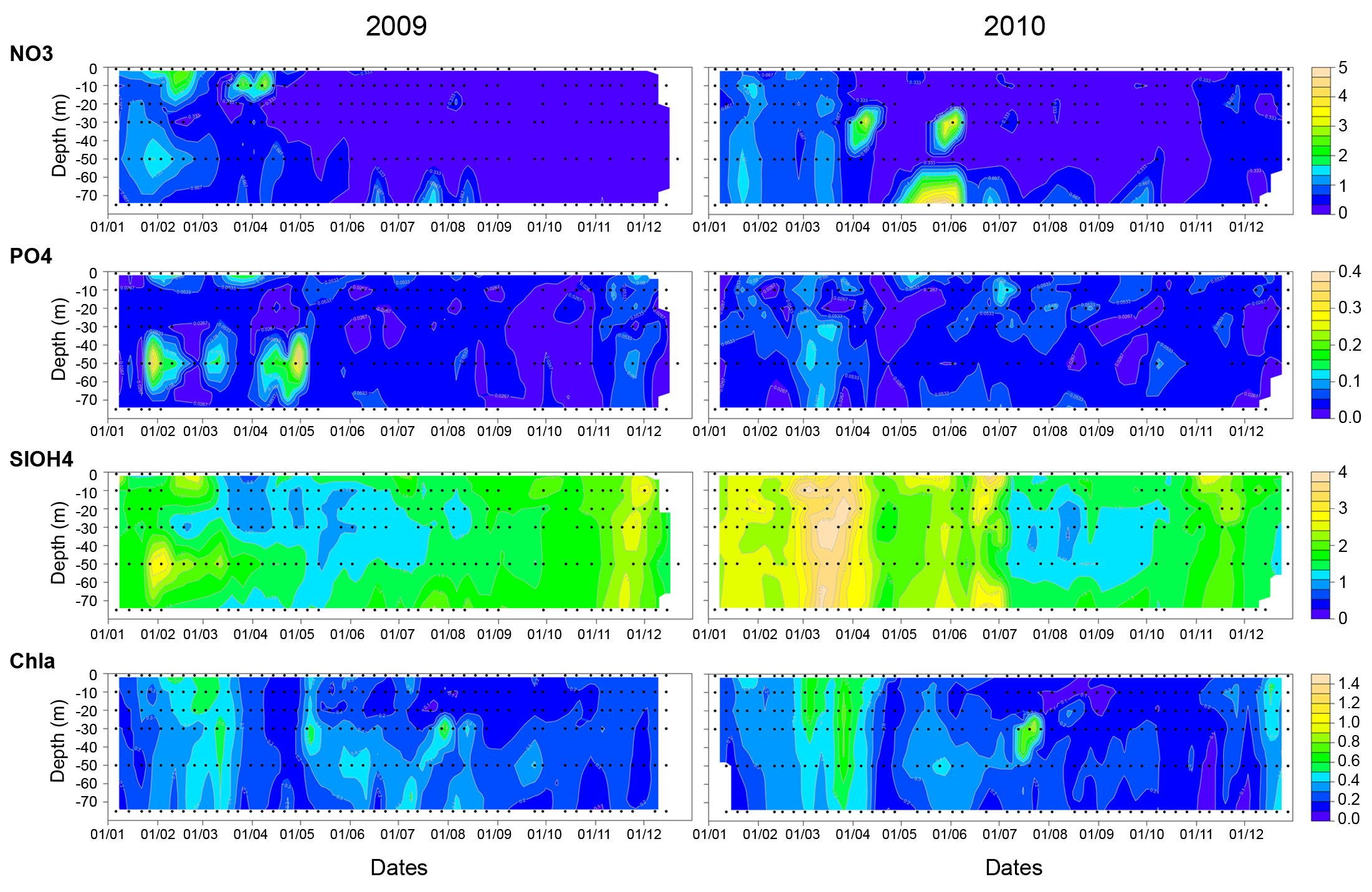

Supplement: S2 Fig — Nitrate (μM NO3-), orthophosphate (μM PO43-), Silicates (μM Si(OH)4) and total Chl a (μg dm-3). (TIF) [file pone.0190121.s002.tif]

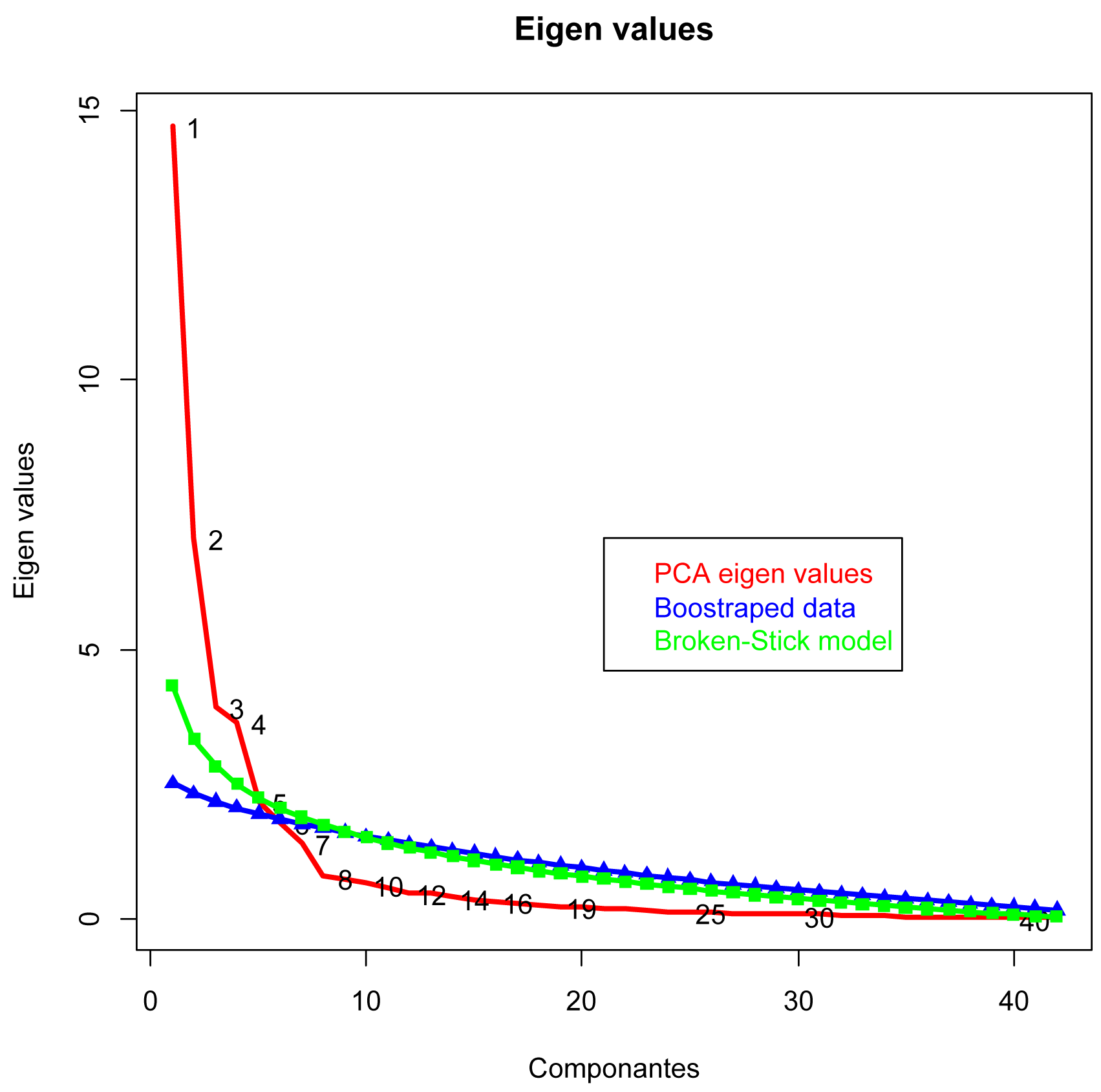

Supplement: S3 Fig — (TIF) [file pone.0190121.s003.tif]

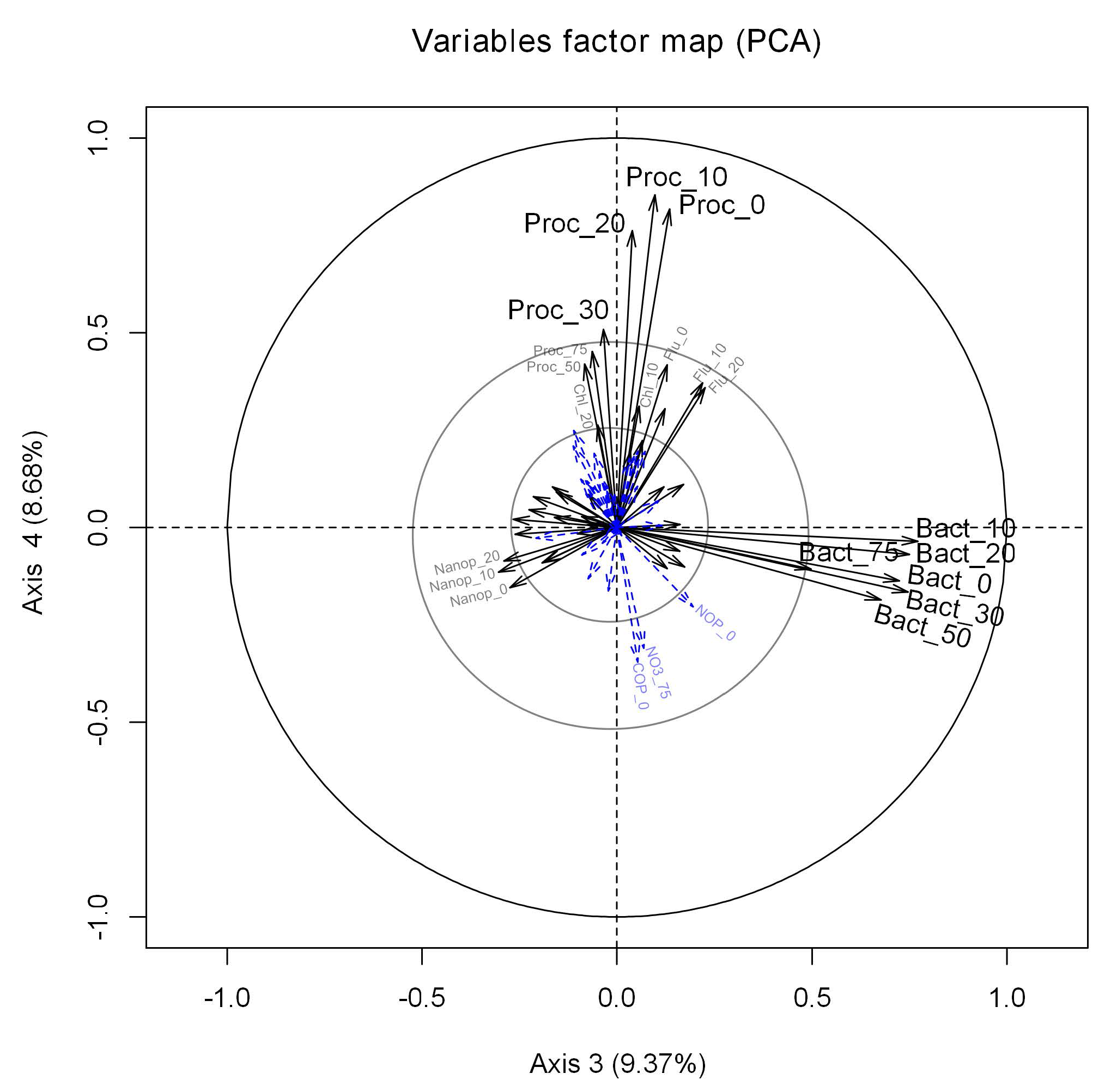

Supplement: S4 Fig — The two orthogonal structures representing HP abundance at all depths and the residual evolution of surface (0-20m) Prochlorococcus abundance. (TIF) [file pone.0190121.s004.tif]
