# Supplementary material for: Variability of ultraplankton composition and distribution in an oligotrophic coastal ecosystem of the NW Mediterranean Sea derived from a two-year survey at the single cell level
Source: PLoS One. 2017 Dec 21;12(12):e0190121. doi: 10.1371/journal.pone.0190121 (PMC5739496; doi:10.1371/journal.pone.0190121)
Supplement: S1 Table — The threshold for significant correlation coefficients were *** P< 0.0001 and relevant (p≥ 0.50); ** P < 0.001 at p = 0.42; * P < 0.05 was at p = 0.23. (DOCX) [file pone.0190121.s005.docx]

S1 Table. Spearman correlation matrix among integrate variables for 2009 and 2010 N=50. The threshold for significant correlation coefficients were ^***^ *P<* 0*.*0001 and relevant (*p≥* 0*.*50); ^**^ *P <* 0.001 at *p* = 0.42 ; ^*^ *P <* 0*.*05 was at *p* = 0.23.

|  |  | T (°C) | S | P (dbar) | Gamma (kg.m^-3^) | Radiation (Jm^2^) | NO3^-^ | Si(OH)_4_ | PO_4_^3-^ | Chl.a (µg.L-1) | HP (cells/ml) | HNA  (cells/ml) | LNA  (cells/ml) |
| --- | --- | --- | --- | --- | --- | --- | --- | --- | --- | --- | --- | --- | --- |
| Chl.a  (µg.L-1) | 2009 | *ns* | *ns* | *ns* | *ns* | *ns* | 0.303^*^ | *ns* | 0.225 | 1 | 0.473 ^**^ | 0.303^*^ | *ns* |
|  | 2010 | -0.226^*^ | *ns* | *ns* | -0.278^*^ | - *ns* | 0.331^*^ | 0.542^***^ | 0.413 | 1 | *ns* | *ns* | *ns* |
| HP  (cells/ml) | 2009 | *ns* | 0.32^*^ | 0.346^**^ | *ns* | *ns* | 0.409 | *ns* | 0.283 | 0.473^**^ | 1 | 0.513^***^ | 0.338^*^ |
|  | 2010 | *ns* | *ns* | *ns* | *ns* | *ns* | *ns* | *ns* | *ns* | *ns* | 1 | 0.479 ^**^ | 0.557^***^ |
| PicoE (cells/ml) | 2009 | *ns* | *ns* | *ns* | -0.427^**^ | *ns* | 0.582^***^ | *ns* | 0.351 | 0.775^***^ | 0,620^***^ | 0,386^*^ | *ns* |
|  | 2010 | *ns* | *ns* | 0.253 | -0.41^*^ | -0.263^*^ | 0.39^*^ | 0.723^***^ | 0.436^**^ | 0.828^***^ | *ns* | *ns* | *ns* |
| NanoP (cells/ml) | 2009 | *ns* | *ns* | *ns* | *ns* | *ns* | 0.386^*^ | -0.224 | 0.262 | 0.8^***^ | 0.555^***^ | 0.30^*^ | *ns* |
|  | 2010 | *ns* | *ns* | *ns* | *ns* | *ns* | *ns* | 0.695^***^ | 0.428^**^ | 0.751^***^ | *ns* | *ns* | *ns* |
| Synecho (cells/ml) | 2009 | *ns* | *ns* | *ns* | *ns* | 0.343^*^ | *ns* | -0.559^***^ | *ns* | *ns* | 0.299^*^ | *ns* | 0.34^*^ |
|  | 2010 | 0.237^*^ | 0.253^*^ | *ns* | *ns* | 0.594^***^ | *ns* | *ns* | 0.263 | 0.269^*^ | *ns* | -0,335^*^ | 0,332^*^ |
| Prochloro (cells/ml) | 2009 | *ns* | *ns* | *ns* | 0.650^***^ | 0.292^*^ | -0.489^**^ | *ns* | -0.314 | *ns* | *ns* | *ns* | *ns* |
|  | 2010 | 0.391^*^ | *ns* | *ns* | 0.688^***^ | 0.349^*^ | -0.52^***^ | -0.496^**^ | -0.233 | -0.3^*^ | *ns* | *ns* | *ns* |
